# Association of Computed Tomography Perfusion Parameters with 90-Day Functional Independence After Endovascular Thrombectomy

**DOI:** 10.3390/jcm14207268

**Published:** 2025-10-15

**Authors:** Rahul R. Karamchandani, Liang Wang, Hongmei Yang, Shraddha T. Patel, Karan N. Tarasaria, Dale Strong, Jeremy B. Rhoten, Jonathan D. Clemente, Gary Defilipp, Andrew W. Asimos

**Affiliations:** 1 Department of Neurology, Neurosciences Institute, Wake Forest University School of Medicine, Advocate Health, Charlotte, NC 28203, USA; shraddha.patel@wfusm.edu (S.T.P.); karan.tarasaria@advocatehealth.org (K.N.T.); 2 Clinical Quality Analytics, Advocate Health, Charlotte, NC 28203, USA; liang.wang1@advocatehealth.org (L.W.); hongmei.yang@advocatehealth.org (H.Y.); edwin.strong@advocatehealth.org (D.S.); 3 Neurosciences Institute, Advocate Health, Charlotte, NC 28203, USA; jeremy.rhoten@advocatehealth.org; 4 Charlotte Radiology, Neurosciences Institute, Wake Forest University School of Medicine, Advocate Health, Charlotte, NC 28203, USA; jonathan.clemente@charlotteradiology.com (J.D.C.); gary.defilipp@charlotteradiology.com (G.D.); 5 Department of Emergency Medicine, Neurosciences Institute, Wake Forest University School of Medicine, Advocate Health, Charlotte, NC 28203, USA; andrew.asimos@wfusm.edu

**Keywords:** CT perfusion, cerebral blood volume index, endovascular thrombectomy

## Abstract

**Background/Objectives**: Recently, a novel CT perfusion (CTP) parameter, the compensation index (COMPI; ratio of 4 s delayed perfusion to 6 s delayed perfusion), was shown to correlate more strongly with digital subtraction angiographic collaterals than the cerebral blood volume index (CBVI) and hypoperfusion intensity ratio. **Methods**: We retrospectively analyzed all anterior circulation large vessel occlusion patients treated at multiple thrombectomy centers from January to December 2024 to determine the relationship of COMPI and other CTP parameters with the primary outcome: a 90-day modified Rankin Scale (mRS) score of 0–2. Univariable logistic regression was performed to assess the association between each CTP parameter and the primary outcome in the full cohort and in those achieving endovascular reperfusion (modified treatment in cerebral ischemia 2b-3). Multivariable logistic regression was performed to determine factors independently associated with a 90-day mRS score of 0–2. **Results**: 323 subjects (median age 69 [57–78] years, median of National Institutes of Health Stroke Scale 15 [10–19.5]) were included, of whom 146/302 (48.3%) were functionally independent at 90 days. The COMPI was not associated with the primary outcome in the univariate analysis. CBVI was the only CTP parameter independently associated with a 90-day mRS score of 0–2 in the full cohort (per 0.1-point increase, odds ratio 1.349, 95% confidence interval 1.099–1.655, *p* = 0.004) and in those achieving reperfusion. **Conclusions**: The COMPI was not associated with a 90-day mRS score of 0–2. CBVI was associated with independent neurological function in the full cohort and in reperfused patients, supporting its role as a CTP collateral biomarker and potential risk stratification tool before thrombectomy.

## 1. Introduction

Computed tomography perfusion (CTP) provides valuable information regarding the status of brain tissue prior to endovascular thrombectomy (EVT) for large vessel occlusion (LVO) strokes [1]. Reduced cerebral blood flow represents tissue that is thought to be irreversibly damaged (“core” infarction), while regions of low blood perfusion can help determine the volume that is potentially salvageable (“penumbra”) if reperfusion is achieved. Historically, perfusion parameters were instrumental in determining candidacy for EVT beyond the 6 h time window [2,3] and, more recently, for clinical trial eligibility in patients presenting with a large core infarction [4,5].

In addition to quantifying core and penumbral volumes, CTP metrics correlate with alternate measures of collateral circulation [6,7,8,9], predict infarct growth [8,10,11,12] and hemorrhagic conversion [13], and are associated with functional outcomes after thrombectomy [14,15,16,17,18]. The cerebral blood volume index (CBVI), the ratio of the mean blood volume in hypoperfused tissue to that in normal tissue, has been shown to predict infarct growth [8,10,11,12], hemorrhagic conversion after medium vessel occlusion [13], and 90-day functional outcomes in patients with both anterior [15,16,17] and posterior circulation [14] LVOs. Meanwhile, the hypoperfusion intensity ratio (HIR), the proportion of 10 s delayed perfusion (Tmax > 10 s) to that of 6 s delayed perfusion (Tmax > 6 s), is also associated with infarct growth [10,11,12], computed tomography angiogram (CTA) collaterals [6,9], and functional outcomes [18].

More recently, the compensation index (COMPI), the ratio of 4 s delayed perfusion (Tmax > 4 s) to Tmax > 6 s, was shown to correlate with collaterals of digital subtraction angiography (DSA) better than the CBVI and HIR, independent of age, comorbidities, premorbid function, computed tomography (CT) Alberta Stroke Program Early CT Score (ASPECTS), and intravenous thrombolysis [7]. While this suggests that the COMPI may be a novel and perhaps better marker of collateral circulation, its association with functional outcomes has not been explored. Here, we evaluate the relationship of the COMPI and other CTP parameters with functional outcomes after EVT, as reliable surrogates of collateral circulation can guide prognostic conversations and impact future studies of patients presenting with an LVO.

## 2. Materials and Methods

We studied the association between individual CTP parameters and functional outcomes in this multicenter, retrospective analysis. The primary outcome measure was 90-day modified Rankin Scale (mRS), score 0–2. The study was approved by the local Institutional Review Board (IRB00136658), which waived the requirement for patient/proxy consent, given the retrospective design of the study.

Anterior circulation LVO patients with premorbid mRS 0–2, treated with EVT at a joint commission-sponsored Comprehensive Stroke Center or a Thrombectomy Capable Center from 1 January 2024 through to 31 December 2024 were eligible for study inclusion. Data were abstracted from a prospectively maintained code stroke registry, and included demographics, historical stroke risk factors, and presenting details, including National Institutes of Health Stroke Scale (NIHSS), glucose, proximal location of thrombosis, CT ASPECTS, treatment with intravenous (IV) thrombolysis, and time to skin puncture. The ASPECTS was calculated for each patient by a fellowship-trained, board-certified neuroradiologist. Post-thrombectomy, modified treatment in cerebral ischemia (mTICI) scores were documented by the treating neuro-interventionalist. The CBVI, HIR, and COMPI were collected from pre-thrombectomy CTP imaging with automated software (RAPID AI, version 5.9, Menlo Park, CA, USA). In addition, the ratio of Tmax > 4 s to Tmax > 10 s, which we termed the delayed perfusion index (DPI), was also calculated, given its potential to correlate with collateral circulation and outcomes that were analogous to the COMPI (Table 1).

Baseline characteristics were calculated for the full cohort and reported as counts with percentages, means (standard deviation [SD]), and medians (interquartile range [IQR]). Patient groups were compared by functional independence (90-day mRS 0–2) versus dependence (90-day mRS 3–6), using the Chi-square test, Mann–Whitney U test, or Fisher’s exact test, where appropriate. Statistical significance was defined as *p* < 0.05.

Univariable logistic regression analysis was conducted to evaluate the association of each CTP parameter (COMPI, CBVI, HIR, DPI) with the primary outcome. Multivariable logistic regression analysis was conducted to determine whether each CTP parameter and other significant factors in the univariate analysis (*p* < 0.05) were independently associated with 90-day mRS 0–2.

In patients achieving endovascular reperfusion (post-treatment mTICI 2b-3), univariable logistic regression analysis was separately performed and a multivariable logistic regression model was constructed to identify independent associations with the primary outcome.

We applied multiple imputations using chained equations to impute missing data, including missing outcomes and independent variables. This method assumes that the data are missing at random and generates multiple complete datasets by imputing missing values based on observed data. The imputation model included all variables used in the analysis, to ensure consistency with the analytic model. We generated 30 imputed datasets and combined the results to obtain pooled estimates for both the univariate and multivariate analyses. We also checked imputation diagnostics metrics: relative variance increase, fraction of missing information, and relative efficiency. All analyses were conducted with Stata version 18 (StatCorp, College Station, TX, USA).

## 3. Results

During the study period, 371 patients were treated with EVT, 347 of whom had a pre-treatment, anterior circulation LVO. Of these, 24 had a premorbid mRS score of three or greater, leaving 323 total patients, of whom 302 had available 90-day mRS scores (Table 2). Two hundred and ten subjects were included from the health system’s Comprehensive Stroke Center and 113 from the Thrombectomy Capable Center. Missing data were present for CTP parameters, including 8.36% for Tmax > 6 s volumes, 10.22% for Tmax > 10 s volumes, 13.62% for Tmax > 4 s volumes, 14.24% for HIR, and 15.17% for CBVI. The percentage of missing data for each variable can be seen in Table S1.

Table 2 shows baseline demographics, medical history, presentation details, and CTP parameters for the full cohort. The median age for all subjects was 69 (57–78) years, 159/302 (52.6%) were male, and the median presenting NIHSS score was 15 (10–19.5). Characteristics stratified by independent (mRS 0–2) versus dependent (mRS 3–6) 90-day outcomes are also displayed. A total of 146/302 (48.3%) subjects were functionally independent at 90 days. Significant differences between baseline characteristics were seen for age, premorbid mRS, diabetes, atrial fibrillation, smoking, presenting NIHSS, initial glucose, treatment with IV thrombolysis, time last known well (TLKW) to skin puncture, HIR, and CBVI (Table 2).

The univariate analysis is shown in Table 3. Factors associated with 90-day mRS score 0–2 include age, NIHSS, glucose, premorbid mRS score, diabetes, atrial fibrillation, treatment with IV thrombolysis, and CBVI. The COMPI was not associated with the primary outcome in the univariate analysis (odds ratio [OR] 0.987, 95% confidence interval [CI] 0.902–1.081, *p* = 0.783).

The multivariable analysis for the association between CTP parameters and 90-day functional independence is shown in Table 4 and Tables S2–S4. Among CTP parameters, CBVI was the only candidate CTP parameter that was independently associated with 90-day mRS 0–2 in the full cohort (per 0.1-point increase, OR 1.349, 95% CI 1.099–1.655, *p* = 0.004; Table 4). Age, NIHSS, premorbid mRS, and treatment with IV thrombolysis were also independently associated with 90-day mRS 0–2 (Table 4 and Tables S2–S4).

Among the 276 patients who achieved endovascular reperfusion (mTICI 2b-3), CBVI remained the only CTP parameter significantly associated with functional independence in the multivariable analysis (per 0.1-point increase, OR 1.340, 95% CI 1.094–1.614, *p* = 0.005; Table 5).

To evaluate whether the association between CBVI and functional outcome varied by TLKW to skin puncture, we performed a subgroup analysis stratified by TLKW to skin puncture (<6 h versus ≥6 h) for all subjects. We included an additional interaction term between CBVI and TLKW to skin puncture to our multivariate logistic regression, to test for effect modification. The interaction term between CBVI and time window was not statistically significant (*p* = 0.618), indicating that the effect of CBVI on functional outcome did not differ significantly between the time windows.

Regarding missing data, multiple imputations were performed with 30 imputations. Imputation quality was assessed using variance diagnostics. The variance information (Table 6) shows that our imputation quality was good. Relative efficiency exceeded 0.99 for all variables, demonstrating that the number of imputations used provided excellent precision, relative to infinite imputations. The fraction of missing information ranged from 3.6% (diabetes) to 30.3% (CBVI), indicating low-to-moderate levels of missingness across variables. The relative variance increase was highest for CBVI (42.5%) and first blood glucose level (30.0%), indicating that standard errors for these variables were inflated by approximately 42% and 30%, respectively, due to the missing data. These fractions of missing information values are within acceptable ranges and suggest that the imputation procedure adequately captured the uncertainty associated with missing data.

## 4. Discussion

In this multicenter, retrospective analysis of thrombectomy patients from a prospectively maintained registry, cerebral blood volume index (CBVI) was the only CTP parameter independently associated with a 90-day mRS score of 0–2. The compensation index (COMPI), recently reported to correlate more strongly with DSA collateral scores than CBVI and the hypoperfusion intensity ratio (HIR) [7], did not demonstrate a relationship with functional outcomes; nor did the delayed perfusion index (DPI), a ratio similar to COMPI, though including more prolonged delayed perfusion (Tmax > 10 s; Table 1).

The HIR and CBVI have previously been linked to angiographic collaterals [6,8,9] and infarct growth rate [8,10,11,12]. As CTP parameters, their automated output is advantageous compared with the subjective judgments that are required for the calculation of angiographic collateral scores, which show variable inter-rater reliability [19]. Further, as markers of collateral circulation have been strongly tied to functional outcomes [20], the identification of a potential novel biomarker—more strongly correlated with DSA collaterals than HIR or CBVI, which have been independently associated with functional outcomes in multiple studies [14,15,16,17]—is intriguing. Thus far, the relationship between the COMPI and functional outcomes has not previously been reported, and our findings emphasize the need for future studies replicating the connection between the COMPI and collateral circulation and investigating its connection with functional outcomes in alternate cohorts.

Historically, arterial and venous collateral scores have been tied to functional outcomes [20,21,22]. The cortical vein opacification score, a quantifiable measure of venous collaterals on CTA or DSA, has been independently associated with 90-day mRS in patients presenting with an LVO [21]. DSA collateral scores have similarly been linked with 90-day outcomes in registry [23] and randomized trial [24] cohorts, as have quantifiable measures of arterial collaterals on single- [25,26] and multi-phase [25] CTA. The short time span between 4 and 6 s delayed perfusion may be insufficient to consistently represent an autoregulatory response from vasodilation, which was previously postulated to be the reason that the COMPI correlated with DSA collateral scores [7]. Patient selection may have also played a role in the COMPI not correlating with outcomes, as our cohort was slightly older, had a larger proportion of males, was less frequently treated with IV thrombolysis, and generally had less favorable CBVI, HIR, and COMPI values compared with those reported by Lakhani et al. [7]. However, patient selection in our study was guided by the latest national guidelines and thrombectomy randomized clinical trials [27,28]. Lastly, unmeasured confounders are also an explanatory consideration. As an example, the proportions of common stroke comorbidities are reported in our study, but not in the previous trial correlating the COMPI with DSA collaterals [7].

While the primary purpose of this study was to investigate the relationship between the COMPI and functional independence, it is notable that we have further demonstrated the link between CBVI and outcomes after large vessel occlusion stroke. We have additionally demonstrated the association of the CBVI with functional independence in patients achieving mTICI 2b-3 reperfusion, rather than mTICI 2c-3, as this expands the generalizability of the results to patients with good and not necessarily excellent reperfusion. Our findings add to the existing literature on the prognostic ability of the CBVI, which has previously been independently associated with 90-day functional outcomes after a basilar thrombectomy [14], discharge ambulatory function in patients presenting with ultra-large core (≥100 milliliters) infarctions [15], improved 90-day outcomes in patients being transferred for thrombectomy [16], functional independence in patients treated with a thrombectomy in late time windows [17], and better outcomes one year after anterior circulation EVT [29]. The success of the CBVI in correlating with functional outcomes may lie in its consideration of both arterial and venous collaterals, as it accounts for blood volume in both circulations of hypoperfused tissue compared with normal tissue [7].

Potential future trials investigating expanded indications for thrombectomy may incorporate measures of collaterals, such as the CBVI, as part of the study inclusion. As an example, CBVI > 0.7 was independently associated with a 90-day mRS score of 0–2 in patients transferred for thrombectomy who achieved reperfusion [16]. Another trial demonstrated that patients with CBVI values > 0.7 had better outcomes one year after thrombectomy [29]. The median CBVI in our study in patients achieving 90-day functional independence was 0.8. As collaterals’ “buy time,” slowing the progression from ischemic brain to infarction, a future trial seeking to expand the indications for thrombectomy may use a biomarker such as CBVI for study inclusion. Given our results and those previously reported [16,29], CBVI > 0.7 may be used as an inclusion criterion in a > 24 h thrombectomy trial, or as a cutoff to repeat neuroimaging studies in the case of a protracted interhospital transfer. Pre-thrombectomy CBVI > 0.7 may also be used to guide conversations with family members regarding a range of potential outcomes if successful reperfusion is achieved. Alternatively, a trial investigating a novel neuroprotective agent pre- or post-thrombectomy may use a collateral biomarker for patient selection.

Our study has limitations, namely its retrospective design that subjects it to inherent biases, despite the use of prospectively collected data. This includes a lack of a priori sample size estimation as the sample was based on available registry data. While patients in our cohort were selected for EVT based on updated national guidelines and randomized controlled trials, the additional potential for selection bias exists based on individualized treatment decisions regarding thrombectomy candidacy. Validation of our findings outside of two thrombectomy centers would strengthen the analysis. Additionally, investigation of CTP parameters derived from other commercial software programs, which may show variability in core and penumbral volume calculations [30], would broaden the applicability of our findings. ASPECTS and post-thrombectomy mTICI scores were not calculated by multiple readers and adjudicated, subjecting them to potential biases. Missing data were present, namely for some CTP parameters, including 15% of CBVI values (Table S1). However, the missing data were addressed using imputation, as described in the statistical analysis and presented in the Secion 3: Results, demonstrating that our imputation quality was good.

## 5. Conclusions

In conclusion, the COMPI was not associated with 90-day functional independence in this multicenter, retrospective study of patients treated with EVT. The CBVI was independently associated with good functional outcomes in the full cohort and in those who achieved reperfusion, though our findings are limited by the retrospective study design and the use of a single software platform for CTP post-processing. Future studies are required to replicate the previously reported association between the COMPI and angiographic collaterals, as well as validate the relationship between CBVI and functional outcomes in alternate patient cohorts. A collateral circulation biomarker can be helpful for risk stratification pre- and post-thrombectomy, and as a patient selection tool in future clinical trials.

## Figures and Tables

**Table 1:** Computed Tomography Perfusion Parameters.

CT Perfusion Parameter	Definition
CBVI	Mean blood volume in hypoperfused tissue compared to normal tissue
HIR	Tmax > 10 s divided by Tmax > 6 s
COMPI	Tmax > 4 s divided by Tmax > 6 s
DPI	Tmax > 4 s divided by Tmax > 10 s

CT, computed tomography; CBVI, cerebral blood volume index; HIR, hypoperfusion intensity ratio; Tmax, time-to-maximum; s, seconds; COMPI, compensation index; DPI, delayed perfusion index.

**Table 2:** Baseline Patient Characteristics **^a^**.

	Total *N* = 302	90-Day mRS 0–2 *N* = 146	90-Day mRS 3–6 *N* = 156	*p*-Value ^b^
Demographics				
Age, years, median (IQR)	69.0 (57.0–78.0)	65.0 (54.0–74.0)	71.0 (60.0–82.0)	<0.001
Sex, male, *n* (%)	159 (52.6%)	77 (52.7%)	82 (52.6%)	0.98
Race, *n* (%)				0.81
Black	92 (30.5%)	44 (30.1%)	48 (30.8%)	
White	194 (64.2%)	93 (63.7%)	101 (64.7%)	
Other/unknown	16 (5.3%)	9 (6.2%)	7 (4.5%)	
Medical history				
Pre-morbid mRS, median (IQR)	0 (0–0)	0 (0–0)	0 (0–1)	0.002
Hypertension, *n* (%)	218 (72.2%)	104 (71.2%)	114 (73.1%)	0.72
Hyperlipidemia, *n* (%)	136 (45.0%)	68 (46.6%)	68 (43.6%)	0.60
Diabetes mellitus, *n* (%)	75 (24.8%)	26 (17.8%)	49 (31.4%)	0.006
Coronary artery disease, *n* (%)	57 (18.9%)	27 (18.5%)	30 (19.2%)	0.87
Atrial fibrillation, *n* (%)	82 (27.2%)	30 (20.5%)	52 (33.3%)	0.013
Smoking, *n* (%)	131 (43.4%)	72 (49.3%)	59 (37.8%)	0.044
Presentation Details				
Initial NIHSS, median (IQR)	15.0 (10.0–19.5)	13.0 (8.0–18.0)	17.0 (11.0–21.0)	<0.001
Glucose (mg/dL), mean ± SD	133.4 (49.8)	124.4 (35.4)	142.0 (59.2)	0.002
Site of occlusion, *n* (%)				0.19
Internal carotid artery	72 (23.8%)	31 (21.2%)	41 (26.3%)	
Middle cerebral artery—M1	158 (52.3%)	74 (50.7%)	84 (53.8%)	
Middle cerebral artery—M2	71 (23.5%)	41 (28.1%)	30 (19.2%)	
Other (distal MCA or ACA)	1 (0.3%)	0 (0.0%)	1 (0.6%)	
CT ASPECTS, median (IQR)	10.0 (8.0–10.0)	10.0 (9.0–10.0)	10.0 (8.0–10.0)	0.29
IV thrombolysis, *n* (%)	99 (32.8%)	61 (41.8%)	38 (24.4%)	0.001
TLKW to skin puncture (min), median (IQR)	287.5 (179.0–636.0)	240.0 (171.0–509.0)	325.0 (190.0–751.0)	0.016
CTP Parameters				
HIR, median (IQR)	0.4 (0.2–0.6)	0.4 (0.2–0.6)	0.5 (0.3–0.7)	0.047
CBVI, median (IQR)	0.7 (0.6–0.8)	0.8 (0.7–0.9)	0.7 (0.6–0.8)	<0.001
COMPI, median (IQR)	1.9 (1.5–2.5)	1.9 (1.5–2.4)	1.9 (1.5–2.5)	0.82
DPI, median (IQR)	4.1 (2.6–8.0)	4.4 (2.8–9.0)	3.9 (2.5–7.7)	0.42

^a^ Values are based on observed data; missing values are not imputed. There are 21 missing values for the 90-day mRS. ^b^ *t*-test, Chi-square test, rank-sum test, and Fisher’s exact test were used to calculate the *p*-value. N, total number; IQR, interquartile range; n, number; mRS, modified Rankin Scale; NIHSS, National Institutes of Health Stroke Scale; mg, milligrams; dL, deciliter; SD, standard deviation; MCA, middle cerebral artery; ACA, anterior cerebral artery; CT, computed tomography; ASPECTS, Alberta Stroke Program Early Computed Tomography Score; IV, intravenous; TLKW, time last known well; min, minutes; CTP, computed tomography perfusion; HIR, hypoperfusion intensity ratio; CBVI, cerebral blood volume index; COMPI, compensation index; DPI, delayed perfusion index.

**Table 3:** Univariate Association Analysis for Factors of Interest and 90-Day Functional Independence.

	Odds Ratio	95% Confidence Interval		*p*-Value
Compensation index	0.987	0.902	1.081	0.783
Delayed perfusion index	1.000	1.000	1.001	0.46
CBVI, per 0.1 increase	1.296	1.097	1.530	0.002
Hypoperfusion intensity ratio	0.410	0.143	1.177	0.097
Age	0.969	0.953	0.985	<0.001
Initial NIHSS	0.932	0.899	0.966	<0.001
Initial glucose	0.993	0.987	0.999	<0.001
CT ASPECTS	1.134	0.968	1.328	0.120
Tmax > 4 s volume	0.999	0.998	1.001	0.45
Tmax > 6 s volume	0.999	0.997	1.002	0.597
Tmax > 10 s volume	0.998	0.994	1.001	0.174
TLKW to puncture	1.000	0.999	1.000	0.149
Male sex	1.029	0.656	1.614	0.901
Race, Black as reference				
White	1.011	0.613	1.667	0.966
Other/unknown	1.476	0.515	4.225	0.468
Premorbid mRS, 0 as reference				
1	0.305	0.142	0.654	0.002
2	0.544	0.260	1.140	0.107
Hypertension	0.897	0.540	1.489	0.673
Hyperlipidemia	1.138	0.724	1.789	0.575
Diabetes	0.476	0.277	0.818	0.007
CAD	0.964	0.543	1.709	0.899
Atrial Fibrillation	0.523	0.310	0.880	0.015
Smoking	1.498	0.950	2.361	0.082
IV thrombolysis	2.168	1.313	3.581	0.003
Site of occlusion, ICA as reference				
Middle cerebral artery–M1	1.157	0.660	2.026	0.611
Other (distal MCA or ACA)	1.691	0.875	3.268	0.118

CBVI, cerebral blood volume index; NIHSS, National Institutes of Health Stroke Scale; ASPECTS, Alberta Stroke Program Early Computed Tomography Score; Tmax, time-to-maximum; s, seconds; TLKW, time last known well; mRS, modified Rankin Scale, CAD, coronary artery disease; IV, intravenous; ICA, internal carotid artery; MCA, middle cerebral artery; ACA, anterior cerebral artery.

**Table 4:** Multivariable Logistic Regression Model Including Cerebral Blood Volume Index for the Association with 90-day Functional Independence.

	Odds Ratio	95% Confidence Interval		*p*-Value
CBVI, per 0.1 increase	1.349	1.099	1.655	0.004
Age	0.965	0.945	0.985	0.001
Initial NIHSS	0.937	0.900	0.976	0.002
First blood glucose level	0.995	0.988	1.001	0.114
Pre-morbid mRS, 0 as reference				
1	0.309	0.130	0.736	0.008
2	0.969	0.407	2.307	0.943
Diabetes	0.641	0.331	1.242	0.187
Atrial fibrillation	0.848	0.450	1.599	0.61
IV thrombolysis	3.038	1.689	5.465	<0.001

CBVI, cerebral blood volume index; NIHSS, National Institutes of Health Stroke Scale; mRS, modified Rankin Scale; IV, intravenous.

**Table 5:** Multivariable Logistic Regression Model Including Cerebral Blood Volume Index for the Association with 90-day Functional Independence in Patients Achieving Reperfusion *.

	Odds Ratio	95% Confidence Interval		*p*-Value
CBVI, per 0.1 increase	1.340	1.094	1.641	0.005
Age	0.965	0.944	0.986	0.001
Initial NIHSS	0.936	0.896	0.978	0.003
First blood glucose level	0.994	0.987	1.000	0.054
Pre-morbid mRS, 0 as reference				
1	0.277	0.111	0.691	0.006
2	0.985	0.409	2.370	0.972
Diabetes	0.720	0.361	1.437	0.352
Atrial fibrillation	0.797	0.409	1.553	0.505
IV thrombolysis	2.719	1.508	4.902	0.001

* Modified treatment in cerebral ischemia 2b-3. CBVI, cerebral blood volume index; NIHSS, National Institutes of Health Stroke Scale; mRS, modified Rankin Scale; IV, intravenous.

**Table 6:** Variance Information.

	Within	Between	Total	Relative Variance Increase	Fraction of Missing Information	Relative Efficiency
CBVI, per 0.1 increase	0.0076	0.0031	0.0108	0.4251	0.3026	0.99
Age	0.0001	0	0.0001	0.0807	0.075	0.9975
Initial NIHSS	0.0004	0	0.0004	0.0531	0.0506	0.9983
First blood glucose level	0	0	0	0.3004	0.2338	0.9923
Premorbid mRS						
1	0.1855	0.0104	0.1962	0.058	0.055	0.9982
2	0.1729	0.0221	0.1957	0.1319	0.1173	0.9961
Diabetes	0.1099	0.0039	0.1139	0.0369	0.0356	0.9988
Atrial fibrillation	0.098	0.0065	0.1048	0.0689	0.0647	0.9978
IV thrombolysis	0.0797	0.0097	0.0897	0.1254	0.1122	0.9963

CBVI, cerebral blood volume index; NIHSS, National Institutes of Health Stroke Scale; mRS, modified Rankin Scale; IV, intravenous.

## Data Availability

The original contributions presented in this study are included in the article. Further inquiries can be directed to the corresponding author.

## Supplementary Materials

The following supporting information can be downloaded at: https://www.mdpi.com/article/10.3390/jcm14207268/s1. Table S1: Summary of missing variables; Table S2: Multivariable logistic regression model including compensation index for the association with 90-day functional independence; Table S3: Multivariable logistic regression model including delayed perfusion index for the association with 90-day functional independence; Table S4: Multivariable logistic regression model including hypoperfusion intensity ratio for the association with 90-day functional independence.

## Abbreviations

The following abbreviations are used in this manuscript:

CTP	Computed tomography perfusion
EVT	Endovascular thrombectomy
LVO	Large vessel occlusion
CBVI	Cerebral blood volume index
HIR	Hypoperfusion intensity ratio
Tmax > 10 s	10 s delayed perfusion
Tmax > 6 s	6 s delayed perfusion
CTA	Computed tomography angiogram
COMPI	Compensation index
Tmax > 4 s	4 s delayed perfusion
DSA	Digital subtraction angiography
ASPECTS	Alberta Stroke Program Early Computed Tomography Score
mRS	Modified Rankin Scale
NIHSS	National Institutes of Health Stroke Scale
IV	Intravenous
DPI	Delayed perfusion index
SD	Standard deviation
IQR	Interquartile range
mTICI	Modified treatment in cerebral ischemia
TLKW	Time last known well
OR	Odds ratio
CI	Confidence interval
