# Supplementary material for: Association of Computed Tomography Perfusion Parameters with 90-Day Functional Independence After Endovascular Thrombectomy
Source: J Clin Med. 2025 Oct 15;14(20):7268. doi: 10.3390/jcm14207268 (PMC12565368; doi:10.3390/jcm14207268)
Supplement: Supplementary file 1 [file jcm-14-07268-s001.zip › jcm-3907176-supplementary.pdf]

Table S1. Summary of Missing Variables

Variable	Number Missing	N	Percent Missing
Initial NIHSS	2	323	0.62
Presenting blood glucose	9	323	2.79
CT ASPECTS	7	323	2.17
Tmax > 4s volume	44	323	13.62
Tmax > 6s volume	27	323	8.36
Tmax > 10s volume	33	323	10.22
Hypoperfusion Intensity Ratio	46	323	14.24
Cerebral Blood Volume Index	49	323	15.17
TLKW to skin puncture	2	323	0.62
90-day mRS score	21	323	6.5

N, total number; NIHSS, National Institutes of Health Stroke Scale; ASPECTS, Alberta Stroke Program Early CT Score; Tmax, time-to-maximum; s, seconds; TLKW, time last known well; mRS, modified Rankin Scale

Table S2. Multivariable Logistic Regression Model Including Compensation Index for the Association with 90-day Functional Independence

	Odds Ratio	95% Confidence Interval		P-value
Compensation Index	0.917	0.745	1.342	0.413
Age	0.970	0.951	1.051	0.002
Initial NIHSS	0.913	0.874	1.144	<0.001
First blood glucose level	0.994	0.988	1.012	0.083
Pre-morbid mRS, 0 as reference				
1	0.351	0.149	6.718	0.017
2	0.921	0.388	2.577	0.851
Diabetes	0.702	0.369	2.714	0.283
Atrial Fibrillation	0.938	0.505	1.979	0.840
IV thrombolysis	3.073	1.699	0.589	0.001

NIHSS, National Institutes of Health Stroke Scale; mRS, modified Rankin Scale; IV, intravenous

Table S3. Multivariable Logistic Regression Model Including Delayed Perfusion Index for the Association with 90-day Functional Independence

	Odds Ratio	95% Confidence Interval		P-value
Delayed Perfusion Index	1.000	0.999	1.001	0.955
Age	0.970	0.951	0.988	0.002
Initial NIHSS	0.922	0.885	0.961	<0.001
First blood glucose level	0.994	0.988	1.001	0.075
Pre-morbid mRS, 0 as reference				
1	0.349	0.150	0.813	0.015
2	0.901	0.384	2.116	0.812
Diabetes	0.667	0.352	1.266	0.216
Atrial Fibrillation	0.891	0.481	1.651	0.715
IV thrombolysis	2.905	1.638	5.152	<0.001

NIHSS, National Institutes of Health Stroke Scale; mRS, modified Rankin Scale; IV, intravenous

Table S4. Multivariable Logistic Regression Model Including Hypoperfusion Intensity Ratio for the Association with 90-day Functional Independence

	Odds Ratio	95% Confidence Interval		P-value
Hypoperfusion Intensity Ratio	0.545	0.150	1.973	0.354
Age	0.969	0.950	0.987	0.001
Initial NIHSS	0.929	0.891	0.969	0.001
First blood glucose level	0.994	0.988	1.001	0.085
Pre-morbid mRS, 0 as reference				
1	0.331	0.141	0.775	0.011
2	0.889	0.379	2.084	0.787
Diabetes	0.678	0.356	1.293	0.238
Atrial Fibrillation	0.907	0.488	1.685	0.757
IV thrombolysis	2.972	1.677	5.266	<0.001

NIHSS, National Institutes of Health Stroke Scale; mRS, modified Rankin Scale; IV, intravenous
